# Endothelial Dysfunction and Cardiovascular Risk in Obstructive Sleep Apnea: A Review Article

**DOI:** 10.3390/life12040537

**Published:** 2022-04-05

**Authors:** Miriam Peracaula, Daniela Torres, Paula Poyatos, Neus Luque, Eric Rojas, Anton Obrador, Ramon Orriols, Olga Tura-Ceide

**Affiliations:** 1 Department of Pulmonary Medicine, Dr. Josep Trueta University Hospital of Girona, Santa Caterina Hospital de Salt and the Girona Biomedical Research Institute (IDIBGI), 17190 Girona, Spain; mperacaula@idibgi.org (M.P.); dtorress.girona.ics@gencat.cat (D.T.); ppoyatos@idibgi.org (P.P.); nluque@idibgi.org (N.L.); ericrojas.girona.ics@gencat.cat (E.R.); aobrador.girona.ics@gencat.cat (A.O.); 2 Biomedical Research Networking Centre on Respiratory Diseases (CIBERES), 28029 Madrid, Spain

**Keywords:** obstructive sleep apnea, endothelial dysfunction, endothelial progenitor cells, oxidative stress, cardiovascular disease

## Abstract

Obstructive sleep apnea (OSA) is a respiratory condition during sleep caused by repeated pauses in breathing due to upper airway obstruction. It is estimated that OSA affects 30% of the population, but only 10% are well diagnosed due to the absence of a well-defined symptomatology and poor screening tools for early diagnosis. OSA is associated to an endothelial dysfunction inducing several biological responses such as hypoxia, hypercapnia and oxidative stress, among others. OSA also triggers respiratory, nervous, metabolic, humoral and immunity system activations that increase the possibility of suffering a cardiovascular (CV) disease. In this review, we expose different studies that show the relationship between OSA and endothelial dysfunction and its association with CV pathologies like hypertension, and we define the most well-known treatments and their limitations. Additionally, we describe the potential future directions in OSA research, and we report clinical features such as endothelial progenitor cell alterations that could act as biomarkers for the development of new diagnostic tools and target therapies.

## 1. Obstructive Sleep Apnea (OSA)

Obstructive sleep apnea (OSA) is a respiratory disorder during sleep based on repeated pauses in breathing [1]. OSA is an important healthcare concern and one of the most common sleep disorders [1]. The incidence varies significantly among published epidemiological studies with an overall published burden of 4–30% [2]. Despite this high incidence, only 10% of OSA patients are properly diagnosed and treated [3]. Common risk factors for OSA patients include obesity, regional fat distribution (central pattern of obesity), skin-fat fold thickness, male gender and neck circumference (>41 cm for females and >43 cm for males) [4,5].

**Figure 1:**
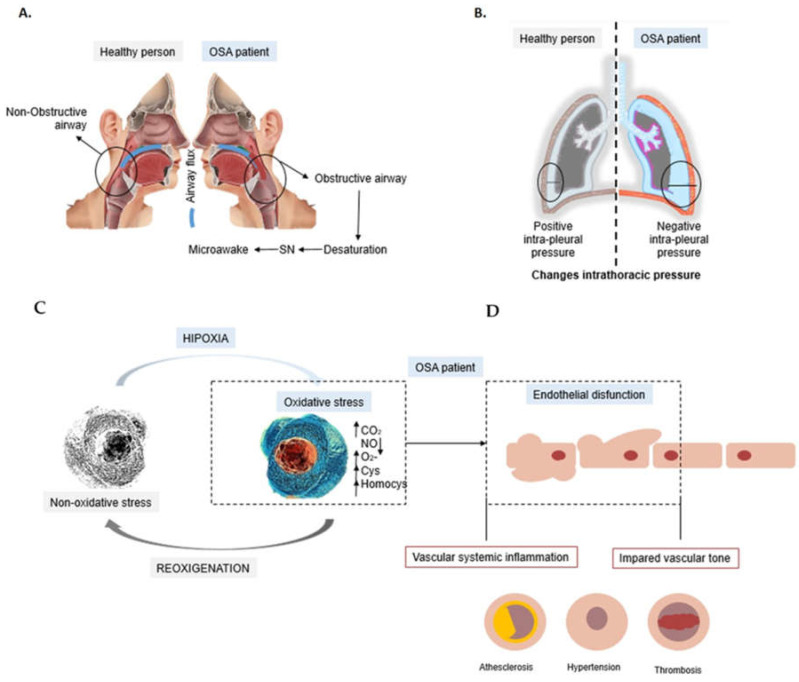
Comparative diagram between healthy people and OSA patients of the most characteristic physical and cellular process of obstructive sleep apnoea (OSA). (**A**). The airway inlet is block in OSA patient causing an obstruction which generates a decrease in blood oxygen which activates de nervous system (SN) leading to a micro-awake. (**B**). Difference in intrathoracic pressure between healthy person and OSA patient due to the obstruction of respiratory airways. (**C**). The cycle of reoxygenation is altered due to the obstruction, and ends up with a decrease in nitric oxide (NO), increase in oxidative stress and systemic inflammation. (**D**). The processes in C lead to a dysfunctional endothelium (ED) which can cause an inflammatory response that can produce different cardiac pathologies such as atherosclerosis, hypertension or thrombosis.

OSA is a disorder of repetitive oropharyngeal collapse during sleep [6]. These events are caused by an imbalance between the force that sustains the airway open (activity of its musculature) and the force that attempts to close it (anatomical and physiological factors). This imbalance between muscle forces causes pharyngeal obstruction and generates total or partial closure of the respiratory airways named apnea or hypopnea, respectively (Figure 1A) [7,8].

The occlusion of the respiratory airways causes hypoxemia (reduced blood oxygen saturation), hypercapnia (increased partial pressure of carbon dioxide in the blood), changes in intrathoracic pressure (Figure 1B) and sympathetic activation, leading to various autonomic and hemodynamic responses [9]. During OSA’s respiratory events, intermittent hypoxia (IH) triggers an increase in oxidative stress which plays an important role in the development of atherogenesis, cardiovascular (CV) disease and endothelial dysfunction [9]. This, together with post-apnea/hypopnea reoxygenation, contributes to the production of reactive oxygen species and inflammatory mediators, leading to upper airway and systemic inflammation [9]. Hypoxia also produces sympathetic activation leading to elevated blood pressure, which is maintained after correction of hypoxia (Figure 1C,D) [10].

All these physiological alterations typify the pathophysiology of OSA. If these biological events are maintained or coexist over a long period of time, OSA patients can develop mild or severe endothelial dysfunction that may lead to a high inflammatory response [10]. Accordingly, OSA can progress to serious healthcare consequences such as an increased risk of suffering cardiovascular morbidities and mortality, stroke and metabolic disorders, among others [11].

## 2. Diagnosis and Severity

The diagnosis of patients with OSA remains challenging because there is not a well-defined symptomatology [6]. The most common OSA symptoms are snoring, witnessed apneas, waking up with a choking sensation and daytime symptoms such as excessive sleepiness, non-restorative sleep, fatigue or tiredness and morning headache [12]. Laboratory polysomnography (PSG) is considered the gold standard test to ease and refine the OSA diagnosis. A home sleep apnea test (HSAT) with a screening tool named STOP-Bang test (Table 1) are also used as complementary diagnostic tools. As a result of these tests, an apnea-hypopnea index (AHI) is obtained. Apneas and hypopneas are counted and averaged by hours of sleep with polysomnography, and by hours of recording time with HSAT. Additionally, the AHI is used to measure the severity of OSA, being considered mild when AHI is ≥5 and <15, moderate if AHI is ≥15 and <30 and severe when AHI is ≥30. If the AHI is in the mild range, <15 events per hour, the presence of other signs/symptoms are required to diagnose OSA (e.g., excessive daytime sleepiness, fatigue, unrefreshing sleep, insomnia, snoring, observed apnea, sleep-related quality of life impairment) or a medical or psychiatric disorder (e.g., hypertension, coronary artery disease, atrial fibrillation, congestive heart failure, stroke, diabetes, cognitive dysfunction or mood disorder). Alternatively, an AHI ≥15/h detected by PSG or HSAT is sufficient to make the diagnosis of OSA, despite the absence of the above symptoms or medical comorbidities [13].

## 3. Endothelial Dysfunction in OSA Patients

Endothelial dysfunction is a vascular abnormality that can predict the development of a vascular disease [12,15]. It has been demonstrated that OSA patients, without any prior vascular disorder, present endothelial dysfunction [15,16,17]. Unfortunately, the mechanisms behind the development of endothelial dysfunction in OSA are unknown [15].

The endothelium is a monolayer of endothelial cells which form the inner cellular lining of the blood vessels and the lymphatic system in contact with blood and circulating cells [18]. The endothelium has different functions: it controls the fluidity of blood, the aggregation of platelets, the vascular tone, the regulation of immunology, inflammation, angiogenesis and acts like an endocrine organ [18]. All these functions are essential in order to maintain the endothelium homeostasis. Hence, a healthy endothelium is required to prevent a biological alteration that can lead to the appearance of different CV diseases.

Therefore, when the endothelium is structurally or functionally altered due to external stimulus named clinical risk factors (e.g., genetic susceptibility, obesity, sex, smoking, etc.), or harmful agents named cellular risk factors (e.g., oxidative stress, metabolomics alterations, inflammation, pollution, etc.), it could lead to an endothelial dysfunction [19]. The endothelial dysfunction is characterized by a dysregulation of the endothelium physiological functions causing a decrease in its vasodilator capacity, an increase in proinflammatory and prothrombotic responses and an abnormal modulation of vascular growth [19].

Upon endothelial damage, there is an increase in proinflammatory molecules such as interleukin-1 (IL-1), interleukin-6 (IL-6), tumor necrosis factor alpha (TNF-α) and C-reactive protein (CRP) leading to a proinflammatory endothelium and endothelial dysfunction.

A dysfunctional endothelium is defined by an increase of the expression of cell adhesion molecules (CAMs) such as E-selectin, vascular cell adhesion molecule-1 (VCAM-1) and intercellular adhesion molecule 1 (ICAM-1). These CAMs are transmembrane proteins that promote endothelial dysfunction, and increase cell adhesion and migration of leukocytes [20,21]. CAMs are stimulated by the nuclear transcription factor (NF-kB) which is activated when several cytokines, such as IL-1, bind to their receptors [20,22,23]. CAMs can be also found circulating into the bloodstream due to EC activation and secretion of soluble types of CAMs into blood [20,24].

A dysfunctional endothelium is also characterized by reduced levels of nitric oxide (NO). NO reduction could be due to a response to an elevated CRP level that can downregulate the expression and the bioactivity of the endothelial nitric oxide synthase (eNOS) [20,25]. Additionally, NO reduction could be as a result of the presence of superoxide anions that appear due to an imbalance between ROS synthesis and antioxidant systems [20]. This phenomenon is named oxidative stress [20,26].

These pathophysiology mechanisms are associated with OSA (Table 2) and could contribute to the development of CV events such as systemic hypertension and other CV diseases (Figure 1D) [27]. Unfortunately, the mechanisms behind a reduced NO availability in OSA are not well-defined [28]. However, several studies have shown an association between the presence of OSA and alterations of the levels of eNOS and NO (Table 2). Moreover, in vivo studies using animal models, and studies with OSA patients with a low CV risk [28,29,30] (Table 2), have demonstrated that endothelial dysfunction is the first vascular consequence of OSA.

In OSA patients, apnea or hyperpnea appears as a result of the muscle force that occludes the upper respiratory airway, generating a negative intrapleural pressure (Figure 1B). This causes an alteration in hypoxemia-reoxygenation cycles (HRC) and the presence of hypercapnia. If HRC is prolonged, the formation of cysteine, homocysteine and superoxide (O_2_^−^) free radicals increase (Figure 1C) [32,33], while nitric oxide (NO) levels decrease due to the activation of eNOS uncoupling [17,34]. These phenomena can derive in the obstruction of the upper airway in the respiratory system and the activation of neural, humoral, metabolic and inflammatory mechanisms [27], resulting in sleep fragmentation, and changes in blood pressure and heart rate. It has been reported that these disturbances affect the vascular endothelium causing an endothelium dysfunction, and enhance the probability of suffering a CV disease (Table 3) [35]. Different authors have described an association between these characteristic alterations of OSA with the development of CV pathologies such as hypertension, arrhythmias, atrial fibrillation, tachycardia and heart failure, among others (Table 3).

## 4. Animal Models and In Vitro Cellular Models of OSA

Most studies demonstrating the relationship between OSA, endothelial dysfunction and its influence in developing a CV disease are based on clinical observations (Table 2). A limited number of animal murine models are available to mimic the effects of OSA [42]. Current animal models present an intermittent obstruction in the airway which reproduces the IH characteristics of OSA patients. This intermittent obstruction can be achieved by a tracheostomy procedure [42,43], introducing an inflatable balloon inside the trachea [42,44], using a nasal mask [42,45] or exposing them to nocturnal cycles of a hypoxic gas mixture and ambient air over a number of weeks [42]. Moreover, these obstructions have the potential to model other OSA characteristics like hypercapnia and sympathetic activation.

Other authors have chosen to use in vitro models to study the molecular pathways affected by the IH. To achieve these conditions in vitro, cells are exposed to specific and fluctuated oxygen concentrations representative of the average conditions occurring in most OSA patients. Several studies have shown that OSA’s conditions might be challenging to replicate in vitro, and it might not truly reflect the biological response to OSA. Patients’ variabilities in terms of the depth and length of hypoxia, apnea frequency and the slope of desaturation and saturation cycles have not been often accurately considered. In addition, there are no standardized protocols for an IH pattern [42]. All these difficulties explain the small number of experimental cell culture studies currently published.

## 5. Role of the Endothelial Progenitor Cells (EPCs)

Endothelial progenitor cells (EPCs) are mononuclear cells that are mobilized from the bone marrow or from its vascular niche to the ischemic region or vascular disturbance [11,46,47,48] in response to vascular damage. EPCs have the ability to proliferate, migrate and differentiate into mature endothelial cells and participate in vascular reparation and regeneration [10].

EPCs can be isolated and cultured from different cell populations, and have been characterized by the expression of hematopoietic progenitor cell (HPC) markers such as CD34 and CD133, and by the presence of vascular endothelial growth factor receptor 2 (VEGFR-2), also named kinase insert domain receptor (KDR), which is inherent in vascular endothelial cells [46,49]. However, the definition of EPCs is still controversial [48]. Cells negative for CD45 and positive for CD34 have been identified as suspected EPCs for their ability to form endothelial cell colonies. These cells could not be differenced phenotypically and functionally from mature endothelial cells in culture [46,50]. Likewise, other studies have also shown that CD133 negative mononuclear cells were able to differentiate in culture into endothelial cells that were almost identical to mature endothelial cells [48,50,51]. Despite the fact that the phenotypic description of an EPC is still under debate, cumulative data evidence the essential role of EPCs in the repair of vascular damage and endothelial homeostasis.

When an alteration in the endothelium occurs, growth factors and cytokines stimulate the recruitment and mobilization of EPCs [52], facilitating the neovascularization of the endothelial damaged areas [15,53]. Endothelial cells migrate to areas of endothelial denudation in response to ischemic stimulus. Hypoxia-inducible factor 1-alpha (HIF-1α) and stroma-derived factor 1 (SDF-1) are well-known cytokines and biological factors involved in EPC mobilization that are rapidly activated to initiate the pro-angiogenesis process.

Growth factors and cytokines such as C-X-C chemokine receptor type 4 (CXCR4) and VEGFR2 interact with EPCs and facilitate their recruitment and mobilization from the bone marrow to the ischemic areas. EPCs use their angiogenic capabilities to repair the affected areas [53]. Hence, EPCs are essential for the maintenance of the vascular homeostasis and the repair process of the endothelium. A deficiency in the number of EPCs, or a fault in its biological function, can also cause endothelial dysfunction [54], which can result in a vascular pathology [55].

It has been demonstrated that the number and the functional activity of EPCs is affected by different CV risk factors such as hypertension, obesity, hypercholesterolemia, diabetes, smoking and also by physiological conditions such as age, sex and physical activity [56,57]. Different studies have reported a significant lower percentage of EPCs in OSA patients, free of any other risk factors, compared to healthy control subjects. Additionally, several studies have found a significant correlation between the number of circulating EPCs and the degree of the apnea-hypopnea index in OSA patients [15,58,59]. In particular, a negative correlation between the number of circulating EPCs with OSA’s severity was detected [59]. Patients with a severe degree of OSA and with a high apnea-hypopnea index had a significantly reduced number of circulating EPCs compared to healthy subjects [15,52]. The number of EPCs was also inversely correlated with the levels of oxidative stress biomarkers, and positively associated with protective antioxidant biomarkers [52]. Interestingly, Wang et al. described that patients with OSA showed an impaired EPC mobilization and an increased EPC apoptosis and dysfunction [52].

## 6. Potential Biomarkers of OSA and CV Alterations

OSA is characterized by recurring episodes of hypoxemia (Figure 1C), also named intermittent hypoxia [52]. The IH is known as the major pathophysiological feature of OSA because it unleashes oxidative stress, systemic inflammation and the activation of the sympathetic nervous system. IH is related to hypercapnia, with changes in intrathoracic pressure and with the presence of arousals that contribute to increase blood pressure. In parallel, IH also translates into repeated periods of desaturation and reoxygenation cycles that increase oxidative stress. This oxidative environment is characterized by an alteration of NO, O_2_^−^ and CO_2_ levels which leads to the activation of systemic inflammatory pathways (Figure 1). The inflammation of the endothelium, together with an impaired vascular tone, increases blood pressure that activates the chemoreflex coders [17,60]. The chemoreflex mediators stimulate the sympathetic nervous system, causing micro-awakenings in OSA patients. Depending on the degree of the described events, OSA patients can evolve to a more severe disease stage.

If systemic inflammation is prolonged in time, the number of circulating EPCs are reduced, and cell apoptosis and functional impairment occurs [61,62]. This suggests that whereas low-grade inflammation could promote EPCs’ mobilization, a high-grade inflammation in an OSA patient has the contrary effect, reducing the number of circulating EPCs [62,63]. Other studies such as Murri et al. and Thum et al. showed how oxidative stress leads to dysfunctional EPCs with a reduced capacity of being mobilized, migrating and interacting with vascular effectors. Additionally, a decrease in the number of EPCs in the vascular system causes a loss of the vascular homeostasis [59,62,64].

Many studies have correlated CV risk factors with a reduction in the number of circulating EPCs [19,46,65]. A decrease in EPCs has become a good predictor of future CV events [46,66]. A reduced number of EPCs leads to a decrease in the endothelium’s repair capacity and causes irreparable endothelial damage with serious CV consequences [67,68,69]. EPCs are not usually found in abundant numbers in the blood flow in healthy individuals, approximately 0.002% from total peripheral blood mononuclear cells [70], but their numbers rapidly increase in response to vascular damage [71]. Accordingly, the number of circulating EPCs in blood could be a useful indicator of endothelial repair capacity and could play an important role in preventing cardiac pathological complications associated with OSA [72].

Additionally, some studies have found a correlation between the presence of CV events or comorbidities such as atherosclerosis or systemic arterial alterations and the presence of dysfunctional EPCs. Consequently, an alteration of EPC function could become a powerful biomarker to prevent different cardiopathologies.

The appearance of other CV diseases such as thrombosis, restenosis or ischemic complications can also be explained by a functional alteration in EPCs. This seems to be more related to an incorrect maturation or recruitment of EPCs instead of the number of circulating EPCs. Non-functional EPCs could lead to a vascular dysfunction characteristic of thrombosis, restenosis and ischemic diseases [71].

Some other clinical features could also be used as predictive markers of the development of CV pathologies in patients with OSA. It has been shown that men have higher probabilities to develop CV complications associated to OSA than women because they usually present higher blood pressure and higher levels of triglycerides in blood. Particularly, young men (<65 years) are the ones that are more affected due to the changes detected in inflammatory cells such as abnormalities in white blood cells and neutrophils [12]. Conversely, despite the fact that women have minor risks to develop a CV pathology generally, women who suffer a CV disease present raised atherosclerotic plaque precursors and dysfunctional monocytes and macrophages.

Therefore, alterations in blood pressure, the number of triglycerides in blood, the presence of atherosclerotic plaque precursors and quantity of neutrophils, monocytes and macrophages, together with the levels of circulating EPCs and their functional alterations, could act as potential CV biomarkers and be used for the development of new targeted therapies [12]. Target therapies can improve the endogenous vascular repair capacity, increasing the number of circulating EPC, restoring dysfunctional EPC, stimulating the mobilization and the recruitment of bone marrow derived EPC and regulating the release of different growth factors, cytokines or inflammatory mediators involved in the vascular reparation. If target therapies achieve some of these objectives, they could prevent OSA’s progression towards a more severe stage and avoid the appearance of lasting CV events.

## 7. Effects of Endothelial Dysfunction in OSA and CV Events

The mechanisms behind the development of an endothelial dysfunction in OSA have not been defined yet, nor any potential biomarkers to identify this dysfunction. Nevertheless, the onset of endothelial dysfunction is used as an indicator of atherosclerosis and as a predictive biomarker of future CV events [19]. The identification of an endothelial dysfunction and the degree of OSA’s severity is essential to establish an early diagnosis and a prognosis of OSA’s disease progression [73]. It has been demonstrated that there is a correlation between the degree of endothelial dysfunction with the severity of OSA and the appearance of pathological CV events [73].

OSA is characterized by an intermittent hypoxia and the presence of hypercapnia producing cyclical alterations of oxygen saturation and desaturation. IH triggers oxidative stress, systemic inflammation and sympathetic activation of the nervous system [52]. This sympathetic activation leads to a negative intrathoracic pressure and nocturnal arousals (Figure 1B), which provoke a cascade of biological altered events that could derive to several CV comorbidities and disease progression [74,75,76].

According to Goldberger et al. and Floras et al. [77,78], several CV comorbidities including stroke, hypertension, end-stage kidney disease, ischemic heart disease, heart failure, atrial fibrillation and hypertrophic cardiomyopathy have been related with OSA, with hypertension being the most well-associated [74,77,78].

## 8. Effect of OSA in Hypertension

The desaturation and reoxygenation cycles in OSA’s patients activate chemoreflex receptors which increase the sympathetic activity of the nervous system [60,76]. This leads to a peripheral vasoconstriction and results in an increase in blood pressure [60,79]. In parallel, the IH stimulates the renin-angiotensin-aldosterone system that as well increases blood pressure and enhances the presence of a systemic hypertension in OSA patients [80]. Recent studies showed that 30–40% of hypertensive patients were diagnosed with OSA (Table 3) [77,80,81]. These results indicate that a significant correlation exists between systemic hypertension and the presence of OSA (Table 3). Accordingly, Peppard et al. also showed a strong relationship between the risk of developing hypertension and the underlying severity of OSA [36]. Patients with mild OSA (defined by a low AHI) and those with severe OSA (defined by high AHI) had three times more probabilities to suffer hypertension than non-OSA patients [36].

## 9. Effect of OSA in Heart Failure and Arrhythmias

OSA is also commonly associated with heart failure (HF). It has been reported that patients with HF are characterized by the presence of chemoreflex sensitivities, hypocapnia and unstable breathing during sleep with a fall in arterial CO_2_ level below the apneic threshold [82].

These alterations generate negative intrathoracic pressures which increase the left ventricular afterload, impair left ventricular relaxation [10,83], reduce cardiac contractility (effecting the systole and diastole movements) and weaken the process of myocyte contraction and relaxation. All these alterations in HF patients are aggravated when the patient is also diagnosed with OSA. OSA patients suffer from night apneas that provoke greater pulmonary capillary wedge pressure causing a more serious drop in the level of CO_2_ in the blood. These low levels of CO_2_ result in an altered contraction and relaxation myocyte movements which increase the probability of having heart failure [4,10].

Similar mechanisms could explain the association between OSA with atrial fibrillations or arrhythmias [73]. Hypoxic events and night arousals, characteristic in OSA patients, activate the sympathetic nervous system leading to CV comorbidities. Although it is not clear how OSA could act as an arrhythmic factor, increasing evidence seems to indicate that patients with OSA are at higher risk to develop atrial fibrillations or arrhythmias [39].

## 10. Effect of OSA in Coronary Syndromes

Several studies have shown a direct relationship between OSA and the presence of an endothelial dysfunction (Table 3). Disorders involving alterations or abnormalities in coagulation factors, endothelial damage, platelet activation and an increase in inflammatory mediators are observed in OSA patients [73]. It has been reported that the presence of OSA is related to oxidative stress in the endothelium and the presence of inflammatory processes. IH induces the release of nitric oxide (NO), reducing the expression of several inflammatory mediators and adhesion molecules, such as transcription nuclear factor-kB, essential for the homeostasis of the endothelium. When these inflammatory mediators and adhesion molecules are decreased, the body’s anti-inflammatory system is hampered. This reduction promotes plaque destabilization and an endothelial dysfunction that can derive in several coronary syndromes like atherosclerosis, thrombus formation, unstable angina and acute myocardial infarction, among others [19].

## 11. Effect of OSA in Metabolic Disorders

OSA has been linked to the presence of metabolic disorders. The inflammatory environment and an elevated oxidative stress in OSA’s patients can result in the loss of important protective biological mechanisms of the endothelium. Cholesterol transport, anti-inflammatory responses, antioxidant and antithrombotic reactions are some of the most important preventive biological mechanisms to balance and guarantee a healthy vascular system. All these CV protective mechanisms are regulated by lipoproteins. Lipoproteins are a combination of lipids and proteins whose principal function is to transport hydrophobic lipids through the bloodstream. It has been shown that the presence of high levels of circulating lipoproteins could become a risk factor for CV diseases [84]. Particularly, it has been demonstrated that patients with high levels of C-reactive protein and lipoprotein(a) presented a higher cardiovascular risk than the ones with healthy levels. In OSA patients, the levels of circulating lipoproteins could be altered by the inflammatory and oxidative environment. Additionally, a loss of lipoprotein function could derive from the development of a metabolic syndrome, defined in patients suffering a combination of some of the following disorders: visceral obesity, arterial hypertension, hyperglycemia and dyslipidemia [73,76,84]. Metabolic syndrome is associated with the risk of developing type 2 diabetes mellitus and CV disease [74,84].

As previously mentioned, the IH present in OSA patients activates the sympathetic neural system which increases the renin-angiotensin-aldosterone pathway. This increase stimulates glucose intolerance resulting in a higher risk to develop diabetes [13,64].

## 12. Therapies for OSA Patients

OSA treatments aim to control the signs and symptoms of the disease, restore sleep quality, normalize AHI, improve oxyhemoglobin saturation and reduce the risk of complications and health costs [2].

It is important to note that all OSA treatments are key and complementary. Within the treatments, hygienic-dietary measures and control/treatment of potentially reversible diseases (e.g., obesity, hypothyroidism, etc.) are essential to all patients with OSA, regardless of their severity.

The most well-known therapy for OSA patients is continuous positive airway pressure (CPAP) [59]. CPAP is the most clinical and cost-effective therapy. CPAP is applied through a nasal mask or full-face mask and provides continuous positive airway pressure while maintaining the patency of upper airway. This reduces the number of obstructive respiratory events avoiding oxygen desaturation and decreasing arousals that produce an improvement in sleep quality [85].

The use of CPAP is recommended for moderate to severe obstructive sleep apnea patients (AHI > 15) or mild sleep apnea patients (AHI 5–15) with important symptomatology such as excessive daytime sleepiness, non-restorative sleep, insomnia, neurocognitive dysfunction or a history of mood disorder, hypertension or cardiovascular disease. An alternative therapy for OSA patients is the use of an oral appliance (OA) which is an orthodontic retainer that supports the jaw in order to keep the airway flux. This option is more often used in mild and moderate OSA patients rather than in severe cases, but it can be prescribed in patients that do not tolerate CPAP therapy [86]. Besides these non-invasive therapies, surgery can also be used [74,87]. Commonly, patients have obstructions of the airway at different levels and this derived to a trend towards multilevel surgery. Surgery treatment is unusual, due to the current controversial criteria that exist between specialists and the low success rate in OSA recoveries [88,89,90,91]. Importantly, surgical indication does not exclude other treatments, nor vice versa [89,90].

Other first level treatments common to OSA patients, such as positional therapy, weight loss and hypoglossal nerve stimulation are also performed. On the other hand, pharmacologic therapy for OSA patients is not recommended as clinical results have not been effective [74]. Overall, to date, OSA’s treatment is primarily aimed to prevent or correct OSA’s risk factors such as obesity, hypothyroidism or reflux disease [27], rather than restore the endothelial dysfunction developed in OSA’s patients.

## 13. Influence of CPAP in OSA Improvement

Some studies have shown a significant decrease in CV mortality and a reduction in the number of CV events in OSA patients treated with CPAP [92,93,94]. It is suggested that regular CPAP treatment results in the reduction of blood pressure due to the decrease in the activation of the sympathetic neural system. CPAP improves saturation and desaturation cycles avoiding the release of an amplified oxidative stress [75]. These improvements seem to be proportional to the number of hours per night of CPAP use [52]. The reduction of blood pressure is more significant in severe OSA patients, and in patients with longer nightly CPAP use. Interestingly, Jelic et al. have demonstrated that for OSA patients who use CPAP for more than 4 hours a day, the number of EPC levels return to their baseline level, similar to healthy individuals. On the other hand, the EPC levels were unchanged in patients with CPAP use for less than 4 hours a day or untreated patients [15,52].

As has been previously mentioned, OSA patients present low levels of circulating EPCs that could initiate or exacerbate the endothelial dysfunction. However, CPAP treatment has a positive effect in increasing the levels of EPCs in the bloodstream. Despite the fact that CPAP treatment does not cure OSA, its usage is significantly beneficial to avoid hypoxic episodes, reduce cellular apoptosis and promote endothelial repair capacity [37]. The importance of the time length of CPAP use in OSA patients is also described in order to see an improvement in OSA’s progression. Wang, et al. demonstrated that the levels of circulating EPCs and oxidative stress markers were reduced in patients after 1 month of CPAP treatment [52].

CPAP treatment has a direct vasoconstrictor effect. Specifically, it has been seen that CPAP treatment significantly reduces the levels of endothelin-1 (long-acting vasoconstrictor peptide), interleukin-6 (IL-6), C-reactive protein (heightened inflammatory states) and erythropoietin (elevated in procoagulant states) compared to untreated OSA patients [46,52,61].

On the other hand, some studies did not show any significant reduction on mortality rates in patients under CPAP treatment compared to controls [18,95]. Future investigations are needed to further understand the role of CPAP treatment in OSA patients and to elucidate alternative treatments that could be commonly applied to all OSA patients regardless of the patient’s severity.

## 14. Future Directions

From the present review, the most emerging needs in the research field of OSA, its clinical consequences and the need to find a more effective diagnosis and treatments have been highlighted. These new investigations are required to reduce the negative outcomes of OSA related complications, improve the patient’s quality of life and diminish the possibility of developing other diseases derived from OSA such as hypertension or other CV events.

In order to achieve these objectives, future research needs to characterize in more detail the relationship between OSA and the development of CV alterations at a clinical and cellular level. This will allow the identification of different clinical biomarkers that would help to make a good prognosis of CV diseases, as well as establish novel and more specific effective therapies.

## 15. Conclusions

In conclusion, future lines of research such as the (i) generation of improved screening tools for early diagnosis; (ii) finding potential biomarkers that allow identifying which OSA patients are more prone to suffer from CV events; (iii) studying the impact of CV events on the quality of life of OSA patients; and (iv) studying the effectiveness of present treatments and the development of novel therapies are in urgent need.

## Figures and Tables

**Table 1:** Table of the screening tool used for diagnosing OSA patients (STOP-Bang score) [14].

Questions		Answer
Does the patient **snore** loudly?	S	Y/N
Does the patient often feel **tired** during the day?	T	Y/N
Has anyone **observed** the patient stop breathing during their sleep?	O	Y/N
Does the patient suffer from high blood **pressure**?	P	Y/N
Does the patient have a **BMI** > 35?	B	Y/N
Is the patient older than 50 (**age**)?	A	Y/N
Has the patient a **neck** circumference of >40 cm?	N	Y/N
Is the patient male (**gender**)?	G	Y/N
**Scoring** Y ≥ 3 → **High risk** of OSA Y < 3 → **Low risk** of OSA		

Definition of abbreviations: OSA = obstructive sleep apnea, BMI = body mass index, Y = yes, N = no.

**Table 2:** Evidence of the relation between OSA and DE in experimental studies. All scientific papers selected have OSA patients with low CV risk status as participants.

Study	Ref.	Method of ED Detected	Participants
Patt, B. T., et al. (2010).	[16]	FMD, Peroxynitrite levels.	N = 14 (N_OSA_ = 7, N_c_ = 7)
Jelic, S., et al. (2008).	[15]	FMD, NOS levels, Phosphorylated eNOS levels, Cyclooxygenase-2 inducible NOS, levels, Nitrotyrosine. Levels, Circulating EPCs.	N = 32 (N_OSA_ = 32, N_c_ = 15)
Khayat, R. N., et al. (2018).	[17]	FMD, AT1 and AT2 receptors, O_2_*^−^* production in ME, NO production in ME.	N = 21 (N_OSA_ = 11, N_c_ = 10)
Varadharaj, S., et al. (2015).	[28]	O_2_^−^ expression and production in ME, NO expression and production in ME.	N = 31 (N_OSA_ = 19, N_c_ = 12)
Gozal, D., et al. (2007).	[31]	Hyperemic test, sCD40L plasma levels, ADMA plasma levels, Nitrotyrosine plasma levels.	N = 32 (N_OSA_ = 26, N_c_ = 8)
Ip, M. S. M., et al. (2004)	[29]	FMD	N = 40 (N_OSA_ = 28, N_c_ = 12)

Definition of abbreviations: Ref = references, FMD = flow mediated dilatation, NOS = nitric oxide synthase, eNOS = endothelial nitric oxide synthase, NO = oxide nitric, EPCs = endothelial progenitors’ cells, AT1 = Angiotensin II type 1, AT2 = Angiotensin II type 2, ME = microcirculatory endothelium, OSA = obstructive sleep apnea. C = control.

**Table 3:** Evidence of the relation between OSA and CV pathologies in different observational studies.

Study	Ref.	OSA and CV Findings	Study Design
Peppard, P. E., et al. (2000).	[36]	Association of OSA and the presence of hypertension over a four year period.	Prospective study (N = 709, N_SDB_ = 709)
Wang, H., Parker, J. D. et al., (2007).	[37]	Association of untreated OSA with an increased risk of HF.	Prospective study (N = 164, N_M-NSA_ = 113, N_untreated_ OSA = 37, N_treated_ = 14)
Sin, D. D., et al. (1999).	[38]	The presence of OSA is common in CHF population.	Retrospective study (N = 450, N_CHF_ = 450)
Mehra, R., et al. (2006).	[39]	Association of severe SDB with complex arrhythmias.	Multicenter longitudinal study (N = 566, N_SDB_ = 228, N_no-SDB_ = 338)
Monahan, K., et al. (2009).	[40]	Association of apneas and hypopneas during sleep with paroxysmal AF, NSVT, nocturnal arrhythmias and cardiac effects.	Multicenter longitudinal study (N = 2816, N_no arrhytmia_ = 2759, N_PAF, NSDVT_ = 57)
Gami, A. S., et al. (2007).	[41]	OSA is recognized as a risk factor for incident AF.	Retrospective study (N = 3542, N_OSA_ = 3542)

Definition of abbreviations: Ref = references, HF = heart failure, M-NSA = mild or no sleep apnea, CHF = congestive heart failure, SDB = sleep disordered breathing, NDVT = Non-sustained ventricular tachycardia, AF = atrial fibrillation/flutter, OSA = Obstructive sleep apnoea, CV = cardiovascular.

## Data Availability

Not applicable.
